# Supplementary material for: The IQGAP-related RasGAP IqgC regulates cell–substratum adhesion in Dictyostelium discoideum
Source: Cell Mol Biol Lett. 2025 Jan 9;30:4. doi: 10.1186/s11658-024-00678-3 (PMC11720917; doi:10.1186/s11658-024-00678-3)
Supplement: Supplementary file 1 — Supplementary material 1. [file 11658_2024_678_MOESM1_ESM.docx]

***CELLULAR & MOLECULAR BIOLOGY LETTERS:***

**The IQGAP-related RasGAP IqgC regulates cell-substratum adhesion in *Dictyostelium discoideum***

Lucija Mijanović^1^, Darija Putar^1^, Lucija Mimica^1^, Sabina Klajn^1^, Vedrana Filić^1^, Igor Weber^1^

^1^Ruđer Bošković Institute, Department of Molecular Biology, 10000 Zagreb, Croatia

Corresponding author: Igor Weber; Igor.Weber@irb.hr

**Supplementary Information**

This file includes:

- Figures S1-S9 with the corresponding captions.
- Table S1 with the corresponding caption.
- Captions to Supplementary Movies S1-S9.
- Captions to Source Data available in the file Mijanovic_et_al_SourceData.xlsx

**Supplementary Figures**


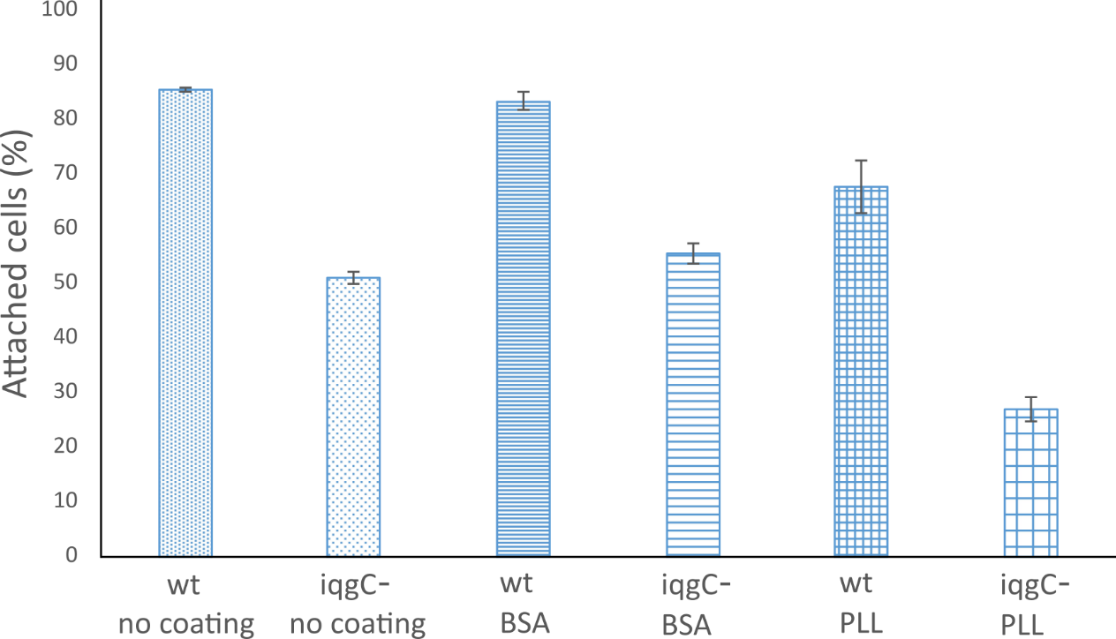


**Fig. S1** Cells lacking IqgC show similar adhesion deficiency compared to wild-type cells on uncoated, BSA-coated and PLL-coated surfaces, as shown by detachment experiments. The number of attached wild-type (wt) and *iqgC*-null (iqgC-) cells was determined after 30 minutes of shaking (mean ± SEM, n (experiments) ≥ 3). There is a significant difference in the percentage of cells that remained attached between wild-type and *iqgC*-null cell lines on all three tested substrata (p < 0.0001, ANOVA followed by Tukey-Kramer test).


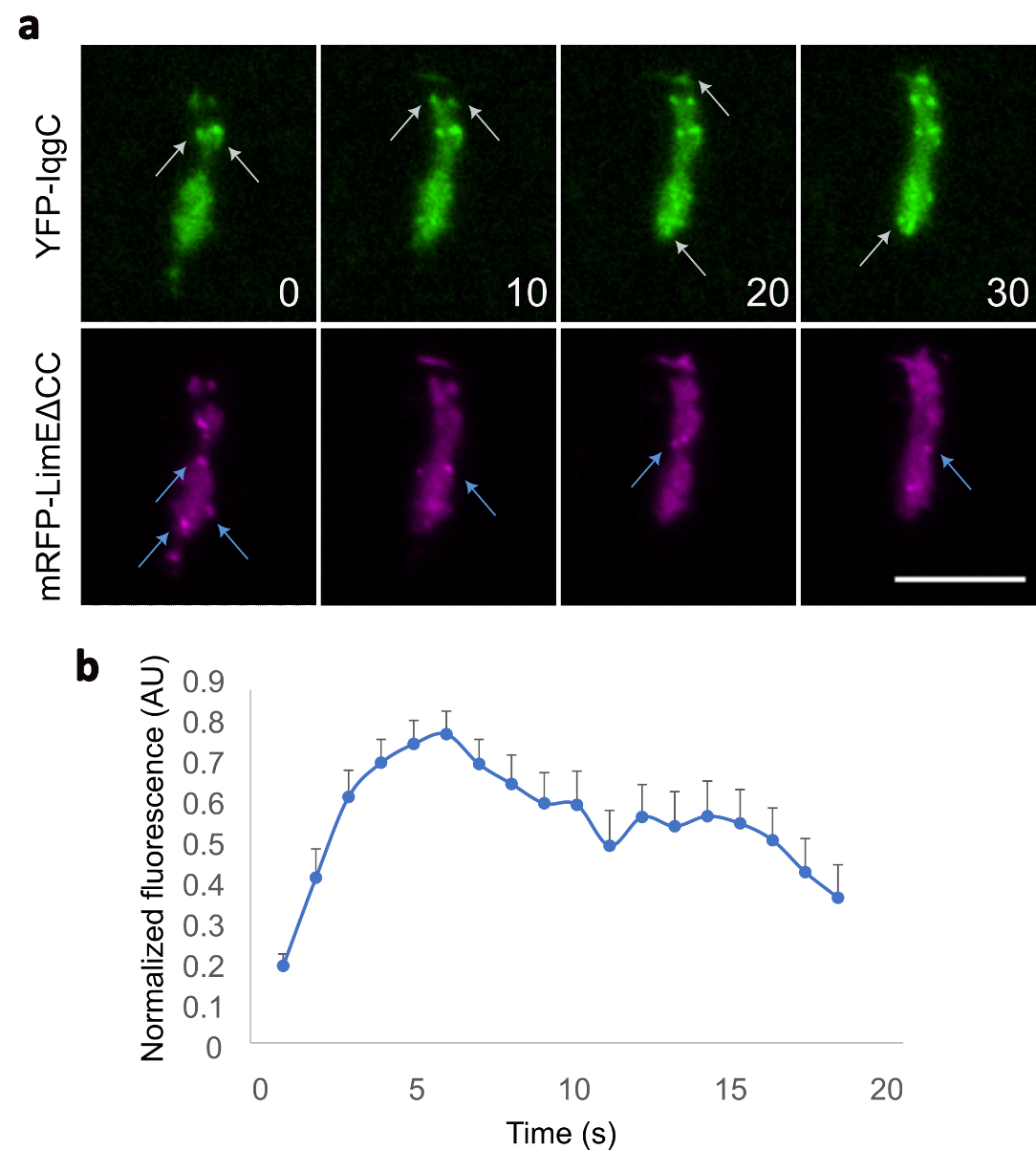


**Fig. S2** IqgC and LimEΔCC do not colocalize in ventral adhesion foci. **a** Dynamics of YFP-IqgC and mRFP- LimEΔCC in ventral foci, as shown by TIRF microscopy. mRFP-LimEΔCC is localized in ventral dot-like structures, which show distinct localization and dynamics in the ventral membrane compared to YFP-IqgC-enriched structures (cell from Supplementary Movie S3). **b** The dynamics of mRFP-LimEΔCC in actin dots (n (dots) = 14; n (exp) = 3).


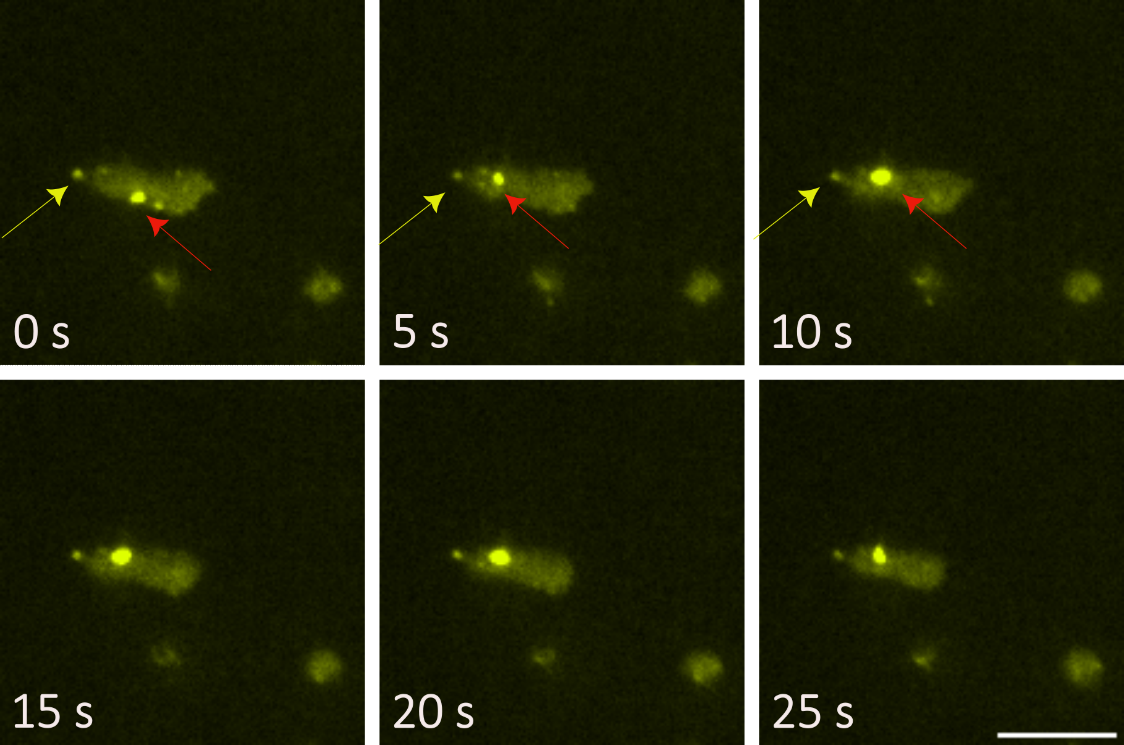


**Fig. S3** A cell expressing YFP-IqgC(RGCt-C) contains a large fluorescent aggregate in the cytoplasm. The aggregates (red arrow) can be distinguished from the adhesion foci (yellow arrow) because the adhesion foci are small and stationary, whereas aggregates are much larger and appear to float around in the cytoplasm. Scale bar, 10 μm.


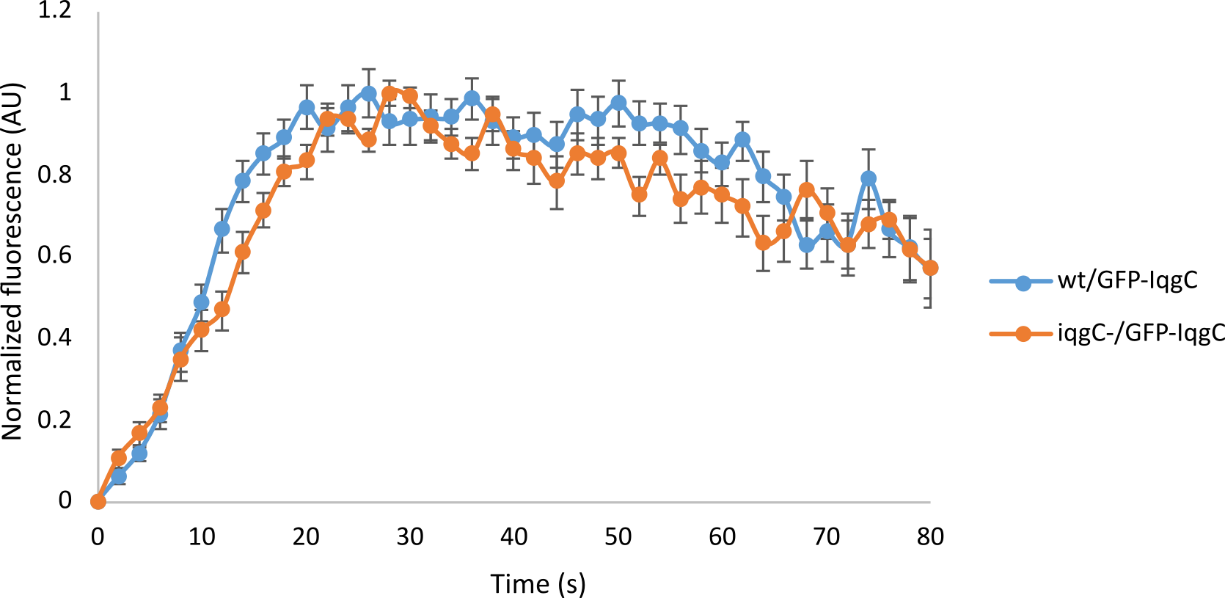


**Fig. S4** Time course of incorporation of GFP-IqgC into adhesion foci is similar in wild-type and *iqgC*-null cells (mean ± SEM, n (experiments) = 2; for wild-type cells: n (foci) = 27, n (cells) = 11; for *iqgC*-null cells: n (foci) = 18, n (cells) = 9).


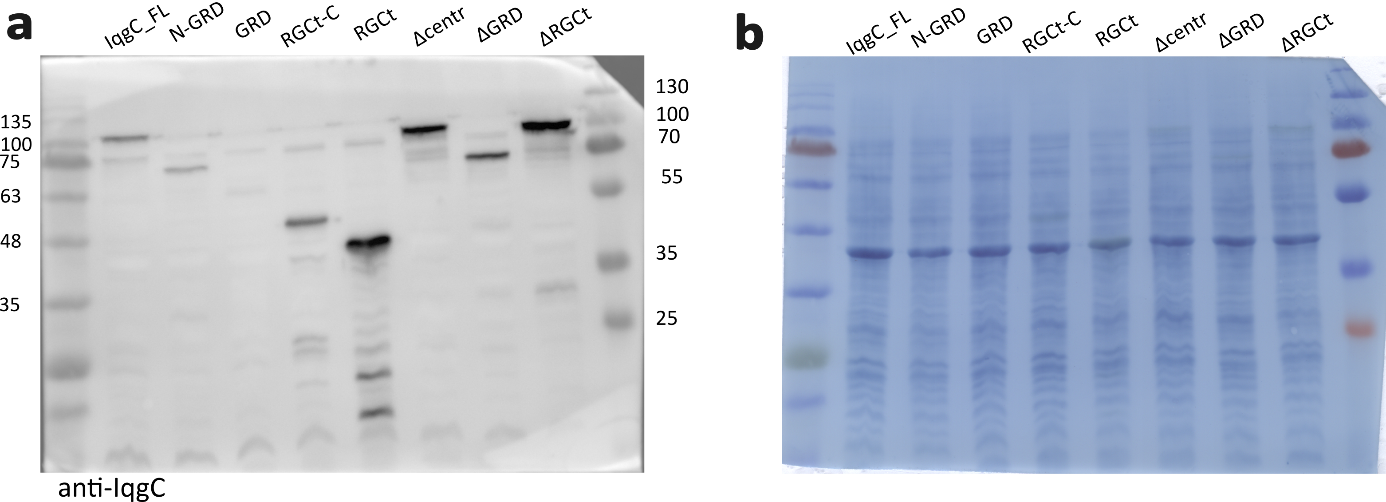


**Fig. S5** Western blot analysis of the expression of YFP-IqgC and YFP-tagged truncated IqgC variants in *iqgC*-null cells. **a** A western blot showing that all variants are expressed and have the correct size (YFP-IqgC_FL 118.8 kDa, YFP-IqgC_N-GRD 85 kDa, YFP-IqgC_GRD 70.9 kDa, YFP-IqgC_RGCt-C 52.9 kDa, YFP-IqgC_RGCt 44.6 kDa, YFP-IqgC_Δcentr 110.7 kDa, YFP-IqgC_ΔGRD 75.7 kDa, YFP-IqgC_ΔRGCt 102 kDa). **b** Amido black staining showing the equal loading of all samples.


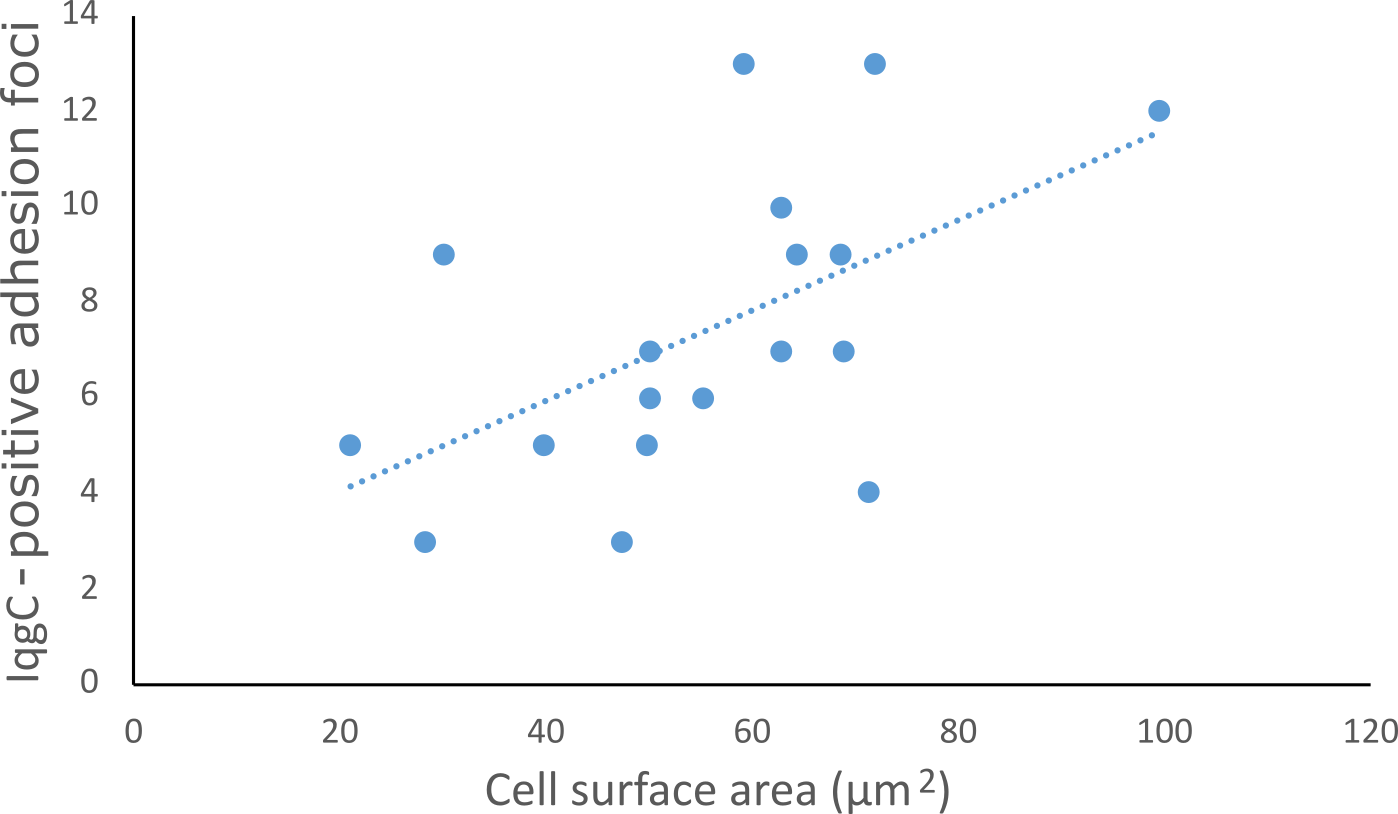


**Fig. S6** Positive correlation between the projected cell surface area and the number of IqgC-positive adhesion foci calculated from the TIRF data of cells co-expressing YFP-IqgC and mRFP-LimEΔCC (Pearson correlation coefficient equal to 0.559, n (cells) = 18, n (experiments) = 3).

**
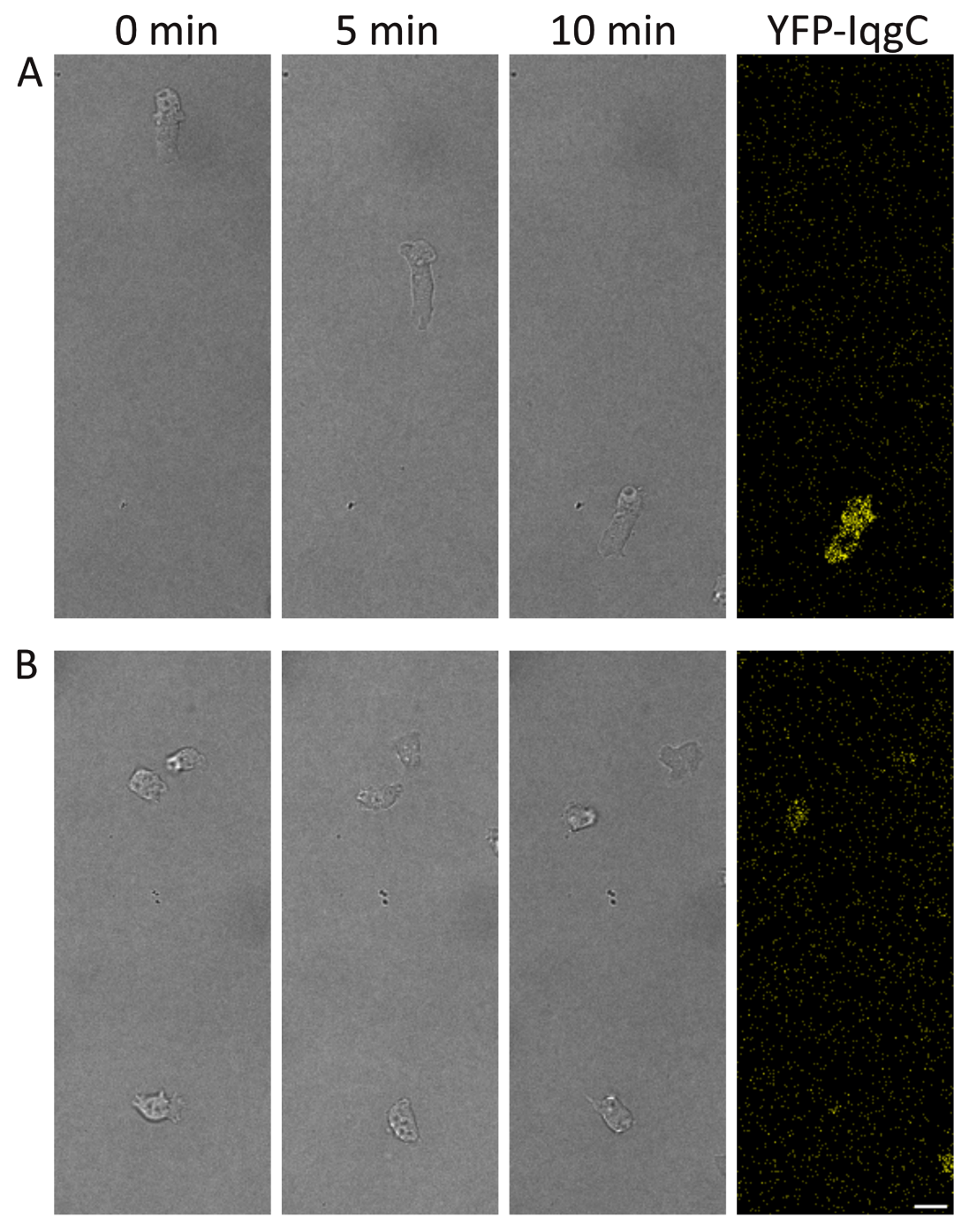
**

**Fig. S7** Examples of different morphologies and motility of (**a**) cells with higher YFP-IqgC expression that are more elongated and move with higher persistence than (**b**) cells with low YFP-IqgC expression. Cells were observed by confocal microscopy while moving randomly on glass. The brightfield image at 10 minutes is also shown in the YFP fluorescence channel. Scale bar, 10 μm.

**
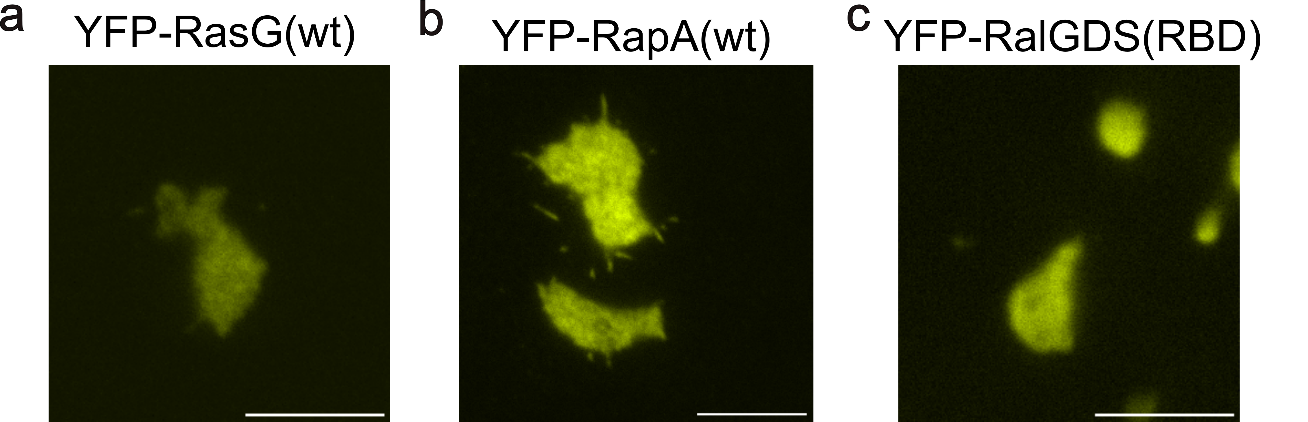
**

**Fig. S8** Uniform localization of (**a**) YFP-RasG(wt), (**b**) YFP-RapA(wt) and (**c**) a probe for active Rap, YFP-RalGDS(RBD) in the ventral membrane during migration on glass in LoFlo medium, imaged by TIRF microscopy. Scale bar, 10 μm.


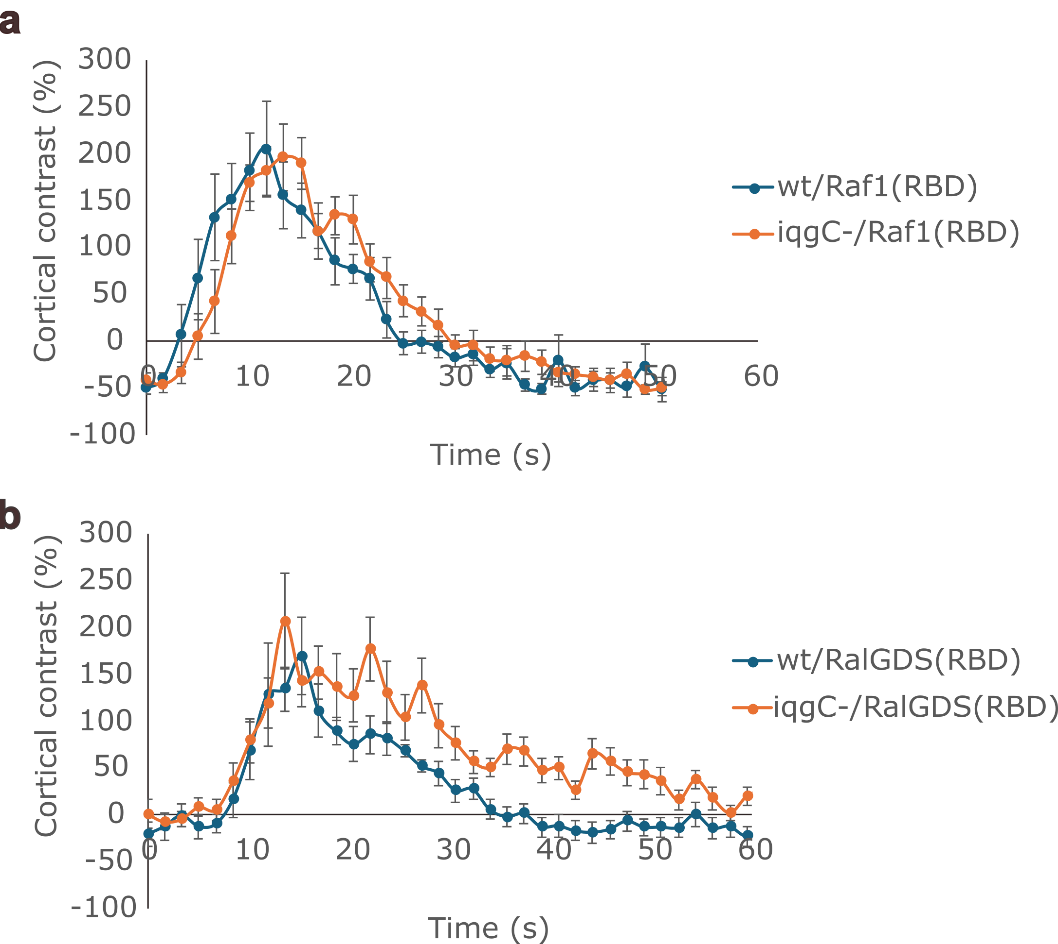


**Fig. S9** Dynamics of activation of Ras (**a**) and Rap (**b**) in wild-type and *iqgC*-null cells expressing fluorescently tagged probes for active Ras (Raf1(RBD)) and Rap (RalGDS(RBD)). Cells were stimulated using 50 μM folic acid (mean ± SEM; for Raf1(RBD): n (experiments) = 2, n (cells) = 12 for wt and 11 for iqgC-; for RalGDS(RBD): n (experiments) = 3, n (cells) = 11 for wt and 12 for iqgC-).

**Supplementary Table**

**Table S1** List of oligonucleotides used in this study.

| Usage | primer | sequence (5' 🡪 3') |
| --- | --- | --- |
| cloning into pDM304, pDM328 and pDM344 vectors | PaxB_BamHI_F1 | ATTGGATCCATGGCAACAAAAGGATTAAATATG |
|  | PaxB_XbaI_R1 | GGCTCTAGATTAAGCAAATAATTTATTATGAC |
|  | LimCC-BglII-F1 | ATTAGATCTATGTCTGCTTCAGTTAAATGTG |
|  | LimCC-SpeI-R1 | AATACTAGTTTAACCAGTTGGTTGACCATC |
|  | RapA_BamHI_F1 | ATTGGATCCATGCCTCTTAGAGAATTCAAAATC |
|  | RapA(FL)_SpeI_R1 | AAACTAGTTTACAATAAAGCACATTTTGATTTAGCTTTGCTTG |
|  | RapA_SpeI_dCAAX_R1 | ATTACTAGTTTATTTTGATTTAGCTTTGCTTGGTG |
|  | RalGDS(RBD)_BglII_F3 | ATTAGATCTATGGACTGCTGTATCATCCGCGTCAG |
|  | RalGDS(RBD)_SpeI_R3 | TAAACTAGTTTACCGCTTCTTGAGGACAAAGTC |
| cloning into pGBKT7 vector | RapA_BamHI-F1 | ATTGGATCCAAATGCCTCTTAGAGAATTCAAAATC |
|  | RapA_PstI-R1 | TAACTGCAGTTATTTTGATTTAGCTTTGCTTGGTG |
| mutagenesis | RapA_G14V_mut-F1 | CGTCGTTTTAGGTTCAGTTGGTGTAGGTAAATCTGC |
|  | RapA_Q65E_mut-F1 | GATACAGCTGGTACTGAAGAATTTACTGCAATGAGAGATC |
|  | RapA_S19N_mut_F3 | GGTTCAGGTGGTGTAGGTAAAAATGCTTTGACTGTGC |
| production of *myoVII* null cells | MyoVII_R1_1 | GAGCAAACAATCATTTGTTCGACTTG |
|  | MyoVII_R1_2 | TAAACAAGTCGAACAAATGATTGTTT |
|  | MyoVII_F1_1 | AGCATCTTTAGCAAAAGCATTATA |
|  | MyoVII_F1_2 | AAACTATAATGCTTTTGCTAAAGA |
|  | MyoVII_seq_fw | CTCATGTTACCTCATATTTTCG |
|  | MyoVII_seq_rev | TCCAATACACCAATAAATGTTGAAT |
| cloning into pGEX-6P-1 vectors | RapA(FL)_SalI_R1 | TAAGTCGACTTACAATAAAGCACATTTTGATTTAGCTTTGC |
|  | GST_N-IqgC_Bam-F1 | ATTGGATCCGATCATTATGGACAATTATTTTATTTAC |
|  | GST_N-IqgC_Sal-R1 | AATGTCGACTTAGAAACGATTTGTGAGTTTC |
|  | GST_C-IqgC_Bam-F1 | ATTGGATCCGTAGATAACATTAAAAAGATATTG |
|  | GST_C-IqgC_Sal-R1 | AATGTCGACTTATCTATCTTCTGGTAC |

**Supplementary Movies**

**Movie S1. A representative wild-type cell expressing YFP-IqgC.** Fluorescently labelled IqgC localizes to ventral adhesion foci during cell migration, as shown by TIRF microscopy (corresponds to Fig 1b). Time is indicated in minutes, scale bar 10 µm.

**Movie S2.** **A representative** **wild-type cell expressing YFP-IqgC** **and mRFP-PaxB**. Fluorescently labelled IqgC and PaxB colocalize to ventral adhesion foci during cell migration, as shown by TIRF microscopy (corresponds to Fig 1c). Time is indicated in minutes, scale bar 10 µm.

**Movie S3. A representative wild-type cell expressing YFP-IqgC and mRFP-LimEdCC.** Fluorescently labelled IqgC and LimEdCC do not colocalize during cell migration, as shown by TIRF microscopy (corresponds to Fig S1). Time is indicated in minutes, scale bar 10 µm.

**Movie S4. A representative *rasG*-null cell expressing YFP-IqgC.** Fluorescently labelled IqgC localizes to ventral adhesion foci during cell migration, as shown by TIRF microscopy (corresponds to Fig 5b). Time is indicated in minutes, scale bar 10 µm.

**Movie S5. A representative *paxB*-null cell expressing YFP-IqgC.** Fluorescently labelled IqgC does not localize to ventral adhesion foci during cell migration, as shown by TIRF microscopy (corresponds to Fig 7c). Time is indicated in minutes, scale bar 10 µm.

**Movie S6. A representative *talA*-null cell expressing YFP-IqgC.** Fluorescently labelled IqgC localizes to ventral adhesion foci during cell migration, as shown by TIRF microscopy (corresponds to Fig 7c). Time is indicated in minutes, scale bar 10 µm.

**Movie S7. A representative *myoVII*-null cell expressing YFP-IqgC.** Fluorescently labelled IqgC localizes to ventral adhesion foci during cell migration, as shown by TIRF microscopy (corresponds to Fig 7c). Time is indicated in minutes, scale bar 10 µm.

**Movie S8. A representative wild-type cell expressing YFP-PaxB.** Fluorescently labelled IqgC localizes to ventral adhesion foci during cell migration, as shown by TIRF microscopy (corresponds to Fig 8b). Time is indicated in minutes, scale bar 10 µm.

**Movie S9. A representative *iqgC*-null cell expressing YFP-PaxB.** Fluorescently labelled IqgC localizes to ventral adhesion foci during cell migration, as shown by TIRF microscopy (corresponds to Fig 8b). Time is indicated in minutes, scale bar 10 µm.

**Source Data Captions**

**Source data 1.** Data used to generate Fig. 1d, e (quantification of fluorescence intensity over time for YFP-IqgC and mRFP-PaxB). The values represent the absolute intensity in 16-bit pixels, measured with the Integrated Density option from the Analyze menu in ImageJ, displayed as RawIntDen (sum of the values of the pixels in the ROI). The background signal corresponds to the value of the first point in the column, which was subtracted from the subsequent values. The highest signal intensity for each measured adhesion focus was set to the value 1, and the remaining values for the same adhesion focus were normalised with respect to this value. The time interval between images is 1 s.

**Source data 2.** Data used to generate Fig. 2b,c (quantification of fluorescence intensity over time for YFP-IqgC variants). Data were analysed as for Source data 1.

**Source data 3.** Data used to generate Fig. 5c,7d (quantification of fluorescence intensity over time for YFP-IqgC in *rasG-*, *talA-*, and *myoVII*-null cells). Data were analysed as for Source data 1.

**Source data 4.** Data used to generate Fig. 8c (quantification of fluorescence intensity over time for YFP-PaxB in AX2 and *iqgC*-null cells). Data were analysed as for Source data 1.

**Source data 5.** Data used to generate Fig. S4 (quantification of fluorescence intensity over time for GFP-IqgC in wild-type and *iqgC*-null cells). Data were analysed as for Source data 1.

**Source data 6.** Data used to generate Fig. S2 (quantification of fluorescence intensity over time for mRFP-LimEdCC). Data were analysed as for Source data 1.

**Source data 7.** Data used to generate Figs. 3 and S6 (quantification of attached cell area and correlation of IqgC-positive adhesion foci and cell surface area). Area in μm^2^.

**Source data 8.** Data used to generate Figs. 1a, 2d, 5a, 7a,8a and S1(measurement of the percentage of cells that remained attached after shaking).

**Source data 9.** Data used to generate Table 1 and Fig. 4 (sorted x and y coordinates for wild-type, *iqgC*-null and IqgC-overexpressing cells in random motility assays and data used to calculate the correlation of YFP-IqgC fluorescence intensity and cell speed/persistence). 44 wild-type and *iqgC*-null cells, as well as 127 IqgC-overexpressing cells were tracked. For correlation analysis, the 50 IqgC-overexpressing cells with the highest YFP-IqgC fluorescence signal were considered.

**Source data 10.** Data used to generate Table 1 and Fig. 4 (sorted x and y coordinates for wild-type and *iqgC*-null cells in chemotaxis assays). For cAMP chemotaxis, 179 wild-type and 60 *iqgC*-null cells were tracked, while for folate chemotaxis, 487 and 394 cells were tracked, respectively.

**Source data 11.** Data used to generate Fig. S9 (dynamics of activation of Ras and Rap GTPases in response to FA stimulation in wild-type and *iqgC*-null cells). For Ras activation, 12 wild-type and 11 *iqgC*-null cells were analysed, while for Rap activation, 11 and 12 cells were analysed, respectively.

**Source data 12.** Data used to generate Fig. 6 (images of uncropped membranes corresponding to the cropped blot images in Fig. 6a-e and data from GAP assays (Fig. 6g), shown as luminescence signals in arbitrary units).
